# Supplementary material for: Psychometric properties of the smartphone addiction scale-short version (SAS-SV) in Honduran university students
Source: PLoS One. 2025 Jul 31;20(7):e0327226. doi: 10.1371/journal.pone.0327226 (PMC12312923; doi:10.1371/journal.pone.0327226)
Supplement: Attachment — Submitted filename: Reviwer Recommendation.pdf [file pone.0327226.s001.pdf]

Dear Editor,

Thank you for the opportunity to review the manuscript titled "Psychometric Properties of the Smartphone Addiction Scale (SAS-SV) in Honduran university students". Given the high prevalence of smartphone addiction, the evaluation of its measurement tools has become very important. Therefore, this study has certain practical significance, but it is recommended that the following issues be considered before publication.

Abstract:

1. Line 30-32: It is recommended to specify the research method part, specifically verify which validity, calculate the relationship with which variables, and give readers more specific and clear information.
2. Line 35-37: "the SAS-SV demonstrated a strong correlation of problematic Internet use (IAT) with depression, anxiety, and stress (DASS-21)". Based on the results of this study, the authors may be trying to express "Results showed strong correlations between SAS-SV and problematic Internet use, depression, anxiety, and stress". In addition, it is recommended not to put the abbreviations of the scales here, because these abbreviations are not the abbreviations of these concepts. For example, problematic Internet use and IAT are not related by full name and abbreviation, which will cause difficulty in understanding for readers who are not familiar with this field. It is recommended to reorganize the language of this section to avoid misunderstanding.

Introduction:

1. Line 49: For academic work, it would be better to cite peer-reviewed journals, government reports, or studies from credible institutions as more authoritative sources.

1. Please address the research gaps more clearly. This study involved the measurement invariance across sex and ages in the results, but limited justification for this. Especially, there is a lack of mention of age in the introduction and discussion section.
2. Line 65-66: The paragraphs lack logical connection.
3. Since previous studies have validated this scale on Spanish adults and proposed cutoff points, and the age of the sample of this study ranged from 17 to 64 years, also adults, what is the innovation of this study? Highlighting the difference between Honduran population and the populations of previous studies may provide more justification for the current study.
4. It is recommended to present the research hypotheses in the order of reliability, validity, questionnaire structure and measurement invariance.

5. The Introduction section should focus on the research subject, reduce the statement of irrelevant information, provide more evidence for the necessity of the research, and enhance the internal logic and coherence of the content.

Method:
1. There is a lack of introduction of sampling technique.
2. There is a lack of introduction of validity and reliability of the instruments used in this study.
3. Please display the statistical analysis section more clearly. The data analysis method was elaborated, which is good for the duplication of the study. However, the presentation of statistical analysis methods is not common. Please refer to other published articles to modify this section and change the “Analysis” to “Statistical analysis”.

Results:
1. The authors removed 263 participants due to the Insufficient effort/potential carelessness, resulting in a sample of 530, which means one-third of the subjects were excluded. Did the author analyze the impact of excluding so many subjects on the sample representativeness and data analysis results?
2. Please display the results according to the research hypothesis.
3. Please present the data results in a more standard way. It is recommended to refer to similar results that have been published.

Discussion:
2. It is recommended to have a more comprehensive and in-depth discussion around the research objectives and results, especially the less than ideal parts of the research results, such as the RMSEA value being higher than the critical value.
3. The introduction mentioned the critical values in previous studies and the high penetration rate of smartphones in the study area. Are these critical values applicable to this study? What is the smartphone addiction status of the subjects in this study? Clarifying these issues can enhance the implications of the study since the authors stated that “identify students with a PSU early and implement appropriate interventions”. (Line 346-347)
4. There is a lake of discussion regarding the results of measurement invariance across sex and ages.
5. It is recommended to highlight the contributions of the research and suggestions for future research.

Reference:
1. Pay attention to the format of references, such as doi, inconsistent capitalization.
2. Some of the references are more than 20 years, please refer to the more

recent references.
