# Supplementary material for: Psychometric properties of the smartphone addiction scale-short version (SAS-SV) in Honduran university students
Source: PLoS One. 2025 Jul 31;20(7):e0327226. doi: 10.1371/journal.pone.0327226 (PMC12312923; doi:10.1371/journal.pone.0327226)
Supplement: Attachment — Submitted filename: 5. Response to reviewers-2024.10.12.docx [file pone.0327226.s002.docx]

Mexico City, Mexico, October 12^th^ 2024

Dear Editor,

We have reviewed and revised the manuscript according to the instructions provided. Below is a summary of the changes made:

1. **Compliance with PLOS ONE’s style requirements**: We have adjusted the manuscript to ensure it meets the journal’s formatting guidelines, including the appropriate file naming conventions. We referred to and followed the templates provided in the links.
2. **Conclusion section**: As requested, we have added a "Conclusion" section to the manuscript.

We have uploaded the updated version of the manuscript through the platform, ensuring that it adheres to the journal's requirements. Should any further adjustments or additional information be needed, please do not hesitate to let us know.

Thank you for your attention, and we remain available for any further queries.

Best regards,

Dr. Guillermo M. Chans

Institute for the Future of Education, Tecnologico de Monterrey, Monterrey, Mexico

E-mail: [guillermo.chans@tec.mx](mailto:guillermo.chans@tec.mx)
