# Supplementary material for: Psychometric properties of the smartphone addiction scale-short version (SAS-SV) in Honduran university students
Source: PLoS One. 2025 Jul 31;20(7):e0327226. doi: 10.1371/journal.pone.0327226 (PMC12312923; doi:10.1371/journal.pone.0327226)
Supplement: Attachment — Submitted filename: 4. Response to reviewers-PLOS ONE 2025.04.06.docx [file pone.0327226.s003.docx]

**Psychometric properties of the smartphone addiction scale (SAS-SV) in Honduran university students**

Dear Reviewers,

Thank you for all the observations and suggestions. They were very useful in improving our manuscript. Below are the descriptions of the corrective actions performed regarding each of your recommendations.

| **Editor** | **Answers** |
| --- | --- |
| (1)  1. Please ensure that your manuscript meets PLOS ONE's style requirements, including those for file naming. The PLOS ONE style templates can be found at  https://journals.plos.org/plosone/s/  file?id=wjVg/PLOSOne_formatting  _sample_main_body.pdf and  https://journals.plos.org/plosone/s/  file?id=ba62/PLOSOne_formatting_sample  _title_authors_affiliations.pdf | Thank you very much for the reminder. We have reviewed the manuscript to ensure it meets all the journal's requirements. |
| (2) Thank you for stating the following in the Acknowledgments Section of your manuscript:  “The authors thank the participants for their involvement in responding to the survey.  The authors acknowledge the financial and technical support of the Writing Lab, Institute for the Future of Education, Tecnologico de Monterrey, Mexico, in producing this work.”  We note that you have provided funding information that is not currently declared in your Funding Statement. However, funding information should not appear in the Acknowledgments section or other areas of your manuscript. We will only publish funding information present in the Funding Statement section of the online submission form.  Please remove any funding-related text from the manuscript and let us know how you would like to update your Funding Statement. Currently, your Funding Statement reads as follows:  “The author(s) received no specific funding for this work.’  Please include your amended statements within your cover letter; we will change the online submission form on your behalf. | Thank you for your feedback. As requested, we have removed the funding-related text from the Acknowledgments section and have updated our Funding Statement accordingly. |
| (3) We note that you have indicated that there are restrictions to data sharing for this study. PLOS only allows data to be available upon request if there are legal or ethical restrictions on sharing data publicly. For more information on unacceptable data access restrictions, please see http://journals.plos.org/plosone/s/data-availability#loc-unacceptable-data-access-restrictions.  Before we proceed with your manuscript, please address the following prompts:  a) If there are ethical or legal restrictions on sharing a de-identified data set, please explain them in detail (e.g., data contain potentially identifying or sensitive patient information, data are owned by a third-party organization, etc.) and who has imposed them (e.g., a Research Ethics Committee or Institutional Review Board, etc.). Please also provide contact information for a data access committee, ethics committee, or other institutional body to which data requests may be sent.  b) If there are no restrictions, please upload the minimal anonymized data set necessary to replicate your study findings to a stable, public repository and provide us with the relevant URLs, DOIs, or accession numbers. For a list of recommended repositories, please see  https://journals.plos.org/plosone/s/recommended-repositories. You also have the option of uploading the data as Supporting Information files, but we would recommend depositing data directly to a data repository if possible.  We will update your Data Availability statement on your behalf to reflect the information you provide. | We have uploaded the dataset to the Tecnológico de Monterrey’s Data Hub, which is public. We added the following text:  Data availability statement  “The data that support the findings of this study are openly available in Tecnologico de Monterrey Data Hub at https://doi.org/10.57687/FK2/DCVIJU.” |
| (4) Please include captions for your Supporting Information files at the end of your manuscript, and update any in-text citations to match accordingly. Please see our Supporting Information guidelines for more information: <http://journals.plos.org/plosone/s/supporting-information>. | We have not included any supporting information files. All citations are up to date. |

| **Reviewer #1** | **Answers** |
| --- | --- |
| Line 30-32: It is recommended to specify the research method part, specifically verify which validity, calculate the relationship with which variables, and give readers more specific and clear information. | Thank you for your valuable feedback. We have restructured the abstract to provide a clearer and more detailed description of the research methods. Specifically, we have explicitly stated the types of validity assessed and clarified the variables used to evaluate these relationships (problematic Internet use, depression, anxiety, stress, and resilience). Additionally, we have ensured that the information is more specific and transparent to enhance the reader’s understanding. |
| Line 35-37: “the SAS-SV demonstrated a strong correlation of problematic Internet use (IAT) with depression, anxiety, and stress (DASS-21)”. Based on the results of this study, the authors may be trying to express “Results showed strong correlations between SAS-SV and problematic Internet use, depression, anxiety, and stress”. In addition, it is recommended not to put the abbreviations of the scales here, because these abbreviations are not the abbreviations of these concepts. For example, problematic Internet use and IAT are not related by full name and abbreviation, which will cause difficulty in understanding for readers who are not familiar with this field. It is recommended to reorganize the language of this section to avoid misunderstanding. | Thank you for your insightful feedback. We have revised the sentence to clarify that the SAS-SV demonstrated strong correlations with problematic Internet use, depression, anxiety, and stress. We have removed the abbreviations (IAT and DASS-21) from this section to ensure better clarity and avoid confusion for readers unfamiliar with these terms. |
| Line 49: For academic work, it would be better to cite peer-reviewed journals, government reports, or studies from credible institutions as more authoritative sources. | Thank you for your suggestion. We have added a new source to complement our reference list. However, to the best of our knowledge, there is currently no official government report or peer-reviewed scientific article that provides data on the global number of smartphone users. That said, both the Statista report and the source we used are widely cited in numerous peer-reviewed articles, which reinforces their credibility. Regarding data specific to Honduras, we refer to government sources such as the National Telecommunications Commission of Honduras, which reports that approximately eight million Hondurans (82.7%) have a mobile phone line, and seven million (71.7%) are mobile Internet subscribers [55]. We appreciate your feedback and will continue to prioritize authoritative sources when available. |
| Please address the research gaps more clearly. This study involved the measurement invariance across sex and ages in the results, but limited justification for this. Especially, there is a lack of mention of age in the introduction and discussion section. | Thank you for your valuable feedback. We have expanded the introduction to better justify the need for analyzing measurement invariance across sex and age. In the revised version, we provide a clearer rationale for the inclusion of these analyses, addressing the relevance of gender and age as demographic factors in understanding the patterns of problematic smartphone use (PSU). Specifically, we now highlight the inconsistent findings in the literature regarding gender differences in PSU and the significant variability in PSU prevalence across different age groups. This additional explanation underscores the importance of testing for measurement invariance to ensure the applicability and validity of the PSU model across different subpopulations.  Line 67:  “However, it is clear that men and women exhibit different patterns of smartphone use: women tend to focus on social and communication functions, while men are more likely to use smartphones for gaming and Internet browsing [26]. These findings suggest that even if differences in overall PSU levels between men and women are not always observed, distinct usage patterns exist. These observed disparities justify the need for gender-based measurement invariance analysis to determine whether the PSU model is applicable and consistent across both populations.  With respect to age, studies indicate that PSU prevalence varies significantly across age groups, being higher among young adults and university students compared to adolescents and children [18]. These findings highlight age as a relevant factor in understanding differences in problematic smartphone use. The variability in usage patterns across life stages points to the importance of conducting age-based measurement invariance analyses [27], to evaluate whether the factors associated with PSU and the model’s structure remain consistent across different age groups. Such analyses provide a more nuanced understanding of how demographic variables influence PSU and offer robust evidence regarding the model’s validity across age groups.  Line 498  “The analysis of DIF, including both uniform and non-uniform DIF, revealed no statistically significant differences in item performance across sex and age groups. Specifically, the χ2 values for both types of DIF did not reach statistical significance, suggesting that the test items function equivalently and consistently across the subgroups evaluated. This finding is particularly important as it reinforces the measurement invariance of the instrument across diverse populations.  The absence of DIF implies that the scale measures the same underlying construct regardless of sex or age, thereby reducing the risk of bias in the results. This evidence of measurement invariance strengthens the instrument’s construct validity, indicating that demographic variables do not systematically influence participants’ responses. Such invariance is essential when generalizing findings across different subpopulations and supports the use of the instrument in diverse research settings without concern that sex- or age-related bias will compromise the interpretation of results.” |
| Line 65-66: The paragraphs lack logical connection. | We have revised the text to enhance the logical connection between the paragraph discussing the prevalence of PSU and the one addressing the terminology used to refer to the phenomenon. Specifically, we added the following sentence to bridge the two sections:  Line 92  "Given its high prevalence and associated negative outcomes, it is relevant to discuss how PSU is conceptualized and defined in the scientific literature."  This addition helps create a clearer flow between the prevalence of PSU and the ongoing debate over its definition, improving the overall coherence of the paragraphs. |
| Since previous studies have validated this scale on Spanish adults and proposed cutoff points, and the age of the sample of this study ranged from 17 to 64 years, also adults, what is the innovation of this study? Highlighting the difference between Honduran population and the populations of previous studies may provide more justification for the current study. | Thank you for your insightful comment. In response, we have added a section highlighting the specific need for validating the Smartphone Addiction Scale-Short Version (SAS-SV) in the Honduran context. While the SAS-SV has been translated into Spanish, we emphasize that this does not guarantee its validity and reliability across all Spanish-speaking contexts due to the geographic and social variability of the Spanish language. Therefore, validating the scale in the Honduran population is crucial, considering the cultural, socioeconomic, and technological differences that may influence the manifestation of problematic smartphone use in this specific population.  Line 142  “The rapid growth of smartphone use in Honduras—particularly among younger populations—has raised increasing concerns about digital addiction and its potential impact on mental health. Cultural factors may also significantly contribute to the development of PSU[59].  In addition, the country's economic context and the growing accessibility of mobile technologies have made smartphones an essential part of daily life. However, the potential negative effects on mental health remain insufficiently studied.  These contextual factors underscore the importance of investigating PSU in Honduras, where the sociocultural and technological landscape may differ significantly from that of other regions. Yet, the absence of validated evidence specific to the Honduran population poses challenges to conducting such research. While the Smartphone Addiction Scale–Short Version (SAS-SV) has been translated into Spanish, this alone does not ensure its validity and reliability across all Spanish-speaking contexts, given the considerable geographic and social diversity of the Spanish language [60].  Thus, validating the SAS-SV in the Honduran context is essential, as cultural, socioeconomic, and technological differences may influence how PSU manifests in this population. Such validation would not only address a significant gap in the literature but also establish a stronger foundation for future research on problematic smartphone use across Central America.” |
| It is recommended to present the research hypotheses in the order of reliability, validity, questionnaire structure and measurement invariance. | Thank you for your valuable feedback. We understand your recommendation to present the research hypotheses in the order of reliability, validity, questionnaire structure, and measurement invariance. However, based on the logical sequence of validity evidence in psychometric research, we have opted for a slightly different order that we believe better reflects the flow of evidence collection: 1) Dimensionality, where we assess the structure of the construct to ensure the instrument measures what it is intended to measure; 2) Reliability, where we evaluate the internal consistency of the scale; 3) Equivalence between groups, ensuring the instrument works consistently across different groups (gender groups and age); and 4) Convergent Validity, where we correlate our scale with other established measures of related constructs. We believe this sequence provides a more logical and natural progression in presenting validity evidence, and we hope this clarifies our approach. We appreciate your understanding. |
| The Introduction section should focus on the research subject, reduce the statement of irrelevant information, provide more evidence for the necessity of the research, and enhance the internal logic and coherence of the content. | We have updated the Introduction section based on your feedback. We focused more on the main topic, included further evidence to highlight the importance of the research, and improved the overall flow and coherence of the content. |
| There is a lack of introduction of sampling technique. | Thank you for your observation. We have clarified the sampling technique in the revised manuscript. |
| There is a lack of introduction of validity and reliability of the instruments used in this study. | Thank you for bringing this to our attention. We have now included the reliability coefficients for each instrument used in the study, including the Cronbach's alpha and McDonald's omega values, to ensure clarity on the internal consistency of the scales employed. |
| Please display the statistical analysis section more clearly. The data analysis method was elaborated, which is good for the duplication of the study. However, the presentation of statistical analysis methods is not common. Please refer to other published articles to modify this section and change the “Analysis” to “Statistical analysis”. | We have made the necessary revisions to the Statistical Analysis section, adjusting the labeling and providing a clearer explanation of the analysis process. We also included an introductory overview of the methods used. |
| The authors removed 263 participants due to the Insufficient effort/potential carelessness, resulting in a sample of 530, which means one-third of the subjects were excluded. Did the author analyze the impact of excluding so many subjects on the sample representativeness and data analysis results? | In the discussion, we mentioned that the exclusion of participants due to insufficient effort or potential carelessness actually strengthens the validity of the study. By removing those who were not fully engaged, we ensure that the data more accurately reflects the target sample—those who actively and seriously participated in the study. This data cleaning process improves the quality of the results and ensures that the analyses are based on more reliable data, which in turn strengthens the internal validity of the study.  Line 541  “Despite these limitations, the study includes a notable strength: the evaluation of response bias due to insufficient effort or careless responding (IE/C). This response pattern has been discussed in the literature for several years [69], and has more recently been recognized as a significant source of error that can compromise the validity of results in both psychometric and non-psychometric research relying on self-reports [67-69]. With the exception of a few recent studies conducted in Spanish-speaking contexts [113-115], methods for detecting IE/C responses remain infrequently used in empirical research with Spanish-speaking samples.  In the present study, although the detection of IE/C resulted in the exclusion of approximately one-third of the original sample, this decision contributed to two important outcomes: (1) it enhanced the internal validity of the findings by minimizing potential distortions, and (2) the proportion of excluded cases was consistent with prevalence rates reported in the literature [116, 117].” |
| Please display the results according to the research hypothesis. | The results have been organized according to the order of the research hypotheses, ensuring a clear and structured presentation of the findings. Thank you for your suggestion. |
| Please present the data results in a more standard way. It is recommended to refer to similar results that have been published. | The order of presentation follows the standard format commonly used in validations, ensuring a clear and structured presentation of the results. |
| It is recommended to have a more comprehensive and in-depth discussion around the research objectives and results, especially the less than ideal parts of the research results, such as the RMSEA value being higher than the critical value. | Thank you for your valuable feedback. In response to your suggestion, we have expanded the discussion section to provide a more comprehensive and in-depth analysis of the research results.  Line 446  “As an additional note, the results for the RMSEA were inconsistent with our conclusion regarding the goodness of fit of the SAS-SV measurement model. However, the inclusion of RMSEA to conclude about the fit of SEM models, although common, is controversial due to the influence of the model’s degrees of freedom [88, 89]. Importantly, all other fit indices supported the conclusion of adequate model fit, thereby providing continued support for the selected measurement model.” |
| The introduction mentioned the critical values in previous studies and the high penetration rate of smartphones in the study area. Are these critical values applicable to this study? What is the smartphone addiction status of the subjects in this study? Clarifying these issues can enhance the implications of the study since the authors stated that “identify students with a PSU early and implement appropriate interventions”. (Line 346-347). | Thank you for your comment. We have followed the procedure used in the validation of the Spanish version of the SAS-SV, which we used to classify the participants. This approach ensures that the classification of smartphone addiction in our study aligns with the established methodology, providing a clear understanding of the participants' status regarding problematic smartphone use. We appreciate your feedback, as it helps clarify these important aspects of the study.  Line 414  “Kwon et al. [42] originally proposed gender-specific cut-off points for identifying problematic smartphone use (PSU): 31 for males and 33 for females (on a scale ranging from 10 to 60). To estimate the prevalence of PSU in our sample, we followed the procedure used in the Spanish and French adaptations of the SAS-SV [56], beginning with an assessment of gender differences in total SAS-SV scores. Since no statistically significant differences were found between males and females in our sample, t(528) = −1.609, p = 0.108, a single average cut-off point of 32 was applied across genders. This approach allowed for a more homogeneous classification of PSU levels among participants.  Using this unified cut-off, 103 students (19.4%) were classified as exhibiting problematic smartphone use. When examined by gender, 18.5% of males and 19.7% of females were classified as PSU users. Statistically significant differences were observed between PSU and non-PSU groups in total SAS-SV scores, t(528) = −32.775, p < .001. Students in the PSU group had a mean score of 39.22 (SD = 6.01), whereas those in the non-PSU group had a mean score of 18.72 (SD = 5.62).” |
| There is a lake of discussion regarding the results of measurement invariance across sex and ages. | Thank you for your comment. In response, we have added a more detailed discussion regarding the results of measurement invariance across sex and age groups. This additional explanation addresses the implications of these findings and explores potential reasons for the lack of significant differences in the functioning of the test items across these groups. We believe this addition enhances the clarity and depth of the discussion on this important aspect of the study.  Line 498  “The analysis of DIF, including both uniform and non-uniform DIF, revealed no statistically significant differences in item performance across sex and age groups. Specifically, the χ2 values for both types of DIF did not reach statistical significance, suggesting that the test items function equivalently and consistently across the subgroups evaluated. This finding is particularly important as it reinforces the measurement invariance of the instrument across diverse populations.  The absence of DIF implies that the scale measures the same underlying construct regardless of sex or age, thereby reducing the risk of bias in the results. This evidence of measurement invariance strengthens the instrument’s construct validity, indicating that demographic variables do not systematically influence participants’ responses. Such invariance is essential when generalizing findings across different subpopulations and supports the use of the instrument in diverse research settings without concern that sex- or age-related bias will compromise the interpretation of results.” |
| It is recommended to highlight the contributions of the research and suggestions for future research. | Thank you for your valuable feedback. In response to your recommendation, we have expanded the discussion section to explicitly highlight the contributions of our research and provide suggestions for future research.  Line 563  “These findings suggest that the SAS-SV can serve as an effective early screening tool for identifying students at risk of PSU, offering valuable insights into the relationship between smartphone use and mental health. Owing to its psychometric robustness, the scale may be integrated into mental health screenings within educational institutions to facilitate the early detection of at-risk individuals and support timely intervention. Early identification and appropriate support may help prevent the escalation of PSU into more severe psychological conditions, such as chronic anxiety, depression, or stress-related disorders.  In conclusion, the SAS-SV emerges as a valuable tool for both research and practice, enabling the early identification of PSU and supporting intervention strategies aimed at mitigating its negative psychological effects. Given the increasing prevalence of smartphone use among young adults—particularly in academic settings—the implementation of validated screening tools such as the SAS-SV may carry significant public health benefits. Their use could promote healthier digital habits and contribute to the improvement of overall mental health in the population.” |
| Pay attention to the format of references, such as doi, inconsistent capitalization. | All article DOIs have been reviewed, and the capitalization of the titles has been standardized. |
| Some of the references are more than 20 years, please refer to the more recent references. | Thank you for your feedback. We understand your concern regarding the age of some of the references. However, we would like to clarify that the older references are essential, as they pertain to the original validation and adaptations of the IAT and DASS-21, which are foundational to the study and must be included for methodological accuracy. Additionally, some references are related to psychometric procedures and statistical methods that remain relevant and widely cited in the field, which is why they are included. |

| **Reviewer #2** | **Answers** |
| --- | --- |
| (1) Abstract:  To enhance the abstract, we recommend incorporating key details aligned with the IMRAC structure (Introduction , Method , results , and conclusion) and the journal's specific guidelines, ensuring a comprehensive and informative summary of the study | Thank you for your valuable suggestion. In response, we have revised the abstract to align with the IMRAC structure (Introduction, Method, Results, and Conclusion) as recommended. We have ensured that the abstract now provides a more comprehensive and informative summary of the study, adhering to the journal's specific guidelines. We believe these adjustments enhance the clarity and organization of the abstract while offering a clearer overview of the study's objectives, methods, findings, and implications. |
| (2) Introduction:  The introduction effectively highlights the importance of studying PSU and the need for validated assessment tools. However, it could be strengthened by:  Adding a sentence or two about the specific context of smartphone use in Honduras or Latin America. Are there any unique factors that make this population particularly relevant to study?  Briefly mentioning the cultural adaptation process of the SAS-SV. Was any adaptation beyond translation necessary? | Thank you for your valuable feedback. In response to your suggestions, we have expanded the introduction to include a more detailed description of the specific context of smartphone use in Honduras. We highlight unique factors such as the rapid growth of smartphone usage, the role of cultural factors, and the increasing concerns about digital addiction, which make this population particularly relevant for studying Problematic Smartphone Use.  Additionally, in the Methods section, we have specified that no changes were necessary beyond translation for the cultural adaptation of the SAS-SV. The Honduran author of the article considered the Spanish version to be appropriate for the local context, and thus, the item wording remained unchanged.  Line 142  “The rapid growth of smartphone use in Honduras—particularly among younger populations—has raised increasing concerns about digital addiction and its potential impact on mental health. Cultural factors may also significantly contribute to the development of PSU[59].  In addition, the country's economic context and the growing accessibility of mobile technologies have made smartphones an essential part of daily life. However, the potential negative effects on mental health remain insufficiently studied.  These contextual factors underscore the importance of investigating PSU in Honduras, where the sociocultural and technological landscape may differ significantly from that of other regions. Yet, the absence of validated evidence specific to the Honduran population poses challenges to conducting such research. While the Smartphone Addiction Scale–Short Version (SAS-SV) has been translated into Spanish, this alone does not ensure its validity and reliability across all Spanish-speaking contexts, given the considerable geographic and social diversity of the Spanish language [60].  Thus, validating the SAS-SV in the Honduran context is essential, as cultural, socioeconomic, and technological differences may influence how PSU manifests in this population. Such validation would not only address a significant gap in the literature but also establish a stronger foundation for future research on problematic smartphone use across Central America.” |
| (3) Participants:  The convenience sampling method is acknowledged as a limitation, but more discussion on the potential biases this may introduce would be beneficial. For example, are students from this particular university likely to be different from other Honduran students in terms of socioeconomic status or access to technology?  Clarify whether participants received course credit or any other form of compensation for their participation. | Thank you for your valuable feedback. In response to your suggestion, we have expanded the discussion on the potential biases introduced by the use of the convenience sampling method. We have addressed how students from this particular university may differ from other Honduran students in terms of socioeconomic status or access to technology, which could influence the generalizability of the findings.  Additionally, we would like to clarify that the information regarding the lack of compensation or course credit for participation was already included in the Procedure section.  Line 521  “This institution primarily serves students from Indigenous communities and has one of the lowest tuition costs among universities in Honduras. These factors may influence participants’ socioeconomic background and access to technology, thereby limiting the generalizability of the results. Future research in Honduras should aim to include more diverse and representative samples to confirm the present findings.” |
| (4) Instruments:  For the SAS-SV, provide more detail on the specific Spanish adaptation used (e.g., who translated it, what changes were made, and whether it has been used in other studies).  For the DASS-21, mention the specific instructions given to participants regarding the time frame they should consider when answering the questions (e.g., "In the past week..."). | Thank you for your valuable suggestions. In response, we have provided more detail regarding the SAS-SV Spanish adaptation in the Methods section. Specifically, we have clarified that we used the existing Spanish adaptation of the SAS-SV, and a Honduran author reviewed the translation to assess if any modifications were needed for the local context. After this review, no changes to the item wording were deemed necessary.  Regarding the DASS-21, we would like to clarify that the information about the time frame for participant responses is already included in the Instruments section. Specifically, it states, "Participants indicated their responses regarding their emotional states over the past week using a four-point Likert scale…". |
| (5) Procedure:  Specify how the online survey was distributed to students (e.g., through university email lists, social media, etc.).  Elaborate on the measures taken to ensure anonymity and confidentiality of the data. | Thank you for your feedback. We have clarified that the questionnaires were distributed via email and that no personal data allowing the identification of participants was collected. This ensures the anonymity and confidentiality of the responses. |
| (6) Analysis:  Explain the rationale for using the ULSMV estimator in the confirmatory factor analysis. Why was this estimator chosen over other alternatives?  Provide more details on the methods used to detect and handle careless responses (e.g., what specific criteria were used to identify participants with insufficient effort?). | We appreciate your comments. In the manuscript, a brief explanation has been added. We have avoided increasing the information density in this section, as we believe the references already cited in the manuscript (References 74 and 75) provide a detailed explanation of the technical arguments for using ULSMV.  We have added the following clarifications regarding careless responses.  Line 239  “Two complementary methods were applied to identify patterns of insufficient effort or carelessness [67, 68]: response invariability and response inconsistency [68].”  Finally, we have added the following explanation:  Line 260  Participants exceeding both thresholds (LS > 7 and D^2^ > χ^2^_cutoff_) were considered to have produced potentially overly consistent or inconsistent responses due to insufficient effort [67-69]. |
| (7) Results:  When reporting the CFA results, include the values of other relevant fit indices (e.g., CFI, TLI, RMSEA) in addition to the chi-square statistic.  In the invariance testing section, report the change in CFI or RMSEA values used to determine whether invariance was supported. | We appreciate your comments. For all models, three commonly reported fit indicators were reported: CFI, RMSEA, and SRMR.  Exactly, we did not use a measurement invariance approach. For this reason, we intentionally used the generic term "Equivalence between groups" to differentiate it from measurement invariance. Among the different methods of equivalence between groups, measurement invariance and Differential Item Functioning (DIF) analysis are the most well-known methods.  To remain consistent with the SEM framework throughout the main analysis, we used MIMIC/RFA-PI, as explained in the methodology. With this method, reporting CFI or RMSEA in MIMIC/RFA-PI is not relevant.  As a clarifying note, in the footnote in Table 4, we added the following: "…None of the tests were statistically significant." |
| (8) Discussion:  The discussion adequately addresses the limitations of the study. However, it could be expanded to:  Discuss the implications of the findings for researchers and practitioners working with Honduran university students. How can the SAS-SV be used to identify and support students with PSU?  Suggest specific directions for future research, such as exploring the relationship between PSU and academic performance or mental health outcomes in this population. | Thank you for your insightful suggestions. In response, we have expanded the Conclusions section to further discuss the implications of our findings for researchers and practitioners working with Honduran university students. We have highlighted how the SAS-SV can be used as a valuable tool for identifying and supporting students with Problematic Smartphone Use, facilitating early intervention strategies. |
| (9) Clinical Utility: Discuss the potential clinical utility of the SAS-SV for identifying students who may be at risk for problematic smartphone use and for developing interventions to address this issue. | Thank you for your valuable feedback. In response, we have expanded the discussion on the clinical utility of the SAS-SV in the Conclusions section. We highlight its potential as an early diagnostic tool to identify students at risk of Problematic Smartphone Use (PSU) and discuss how its incorporation into mental health screenings in educational institutions could facilitate timely interventions. Additionally, we emphasize its role in mitigating the risk of developing more severe psychological issues and promoting healthier digital habits among university students. |
| (10) Tables:  Ensure that all tables are clearly labeled and that all abbreviations are defined in the table footnotes. | We modified the caption of Figure 1:  Fig 1. Information function and reliability for SAS-SV items and test scores. |
| (11) References:  Check the accuracy and completeness of all references. | We reviewed all references. |
